# Magnitude of stunting and its determinants in children aged 6–59 months among rural residents of Damot Gale district; southern Ethiopia

**DOI:** 10.1186/s13104-018-3666-1

**Published:** 2018-08-03

**Authors:** Lamirot Abera, Tariku Dejene, Tariku Laelago

**Affiliations:** 1 Central Statics Agency, Addis Ababa, Ethiopia; 2 Department of Population Studies, Addis Ababa University, Addis Ababa, Ethiopia; 3 Department of Health Information Technology, Hossana College of Health Science, P.O. Box 159, Hossana, Ethiopia

**Keywords:** Rural dwellers, Children, Stunting

## Abstract

**Objective:**

The objective of this study is assessing magnitude of stunting and its predictors among children aged 6–59 months in Damot Gale district, South Ethiopia. Community based cross sectional study was done at Damot Gale district. About 398 children aged 6–59 months were included in the study. Kebele (small administrative unit) and household were chosen by two-phase cluster sample design. Structured questionnaire was used to gather the data. Anthropometric measurement was also used to get the data. SPSS version 20 was used to analysis the data.

**Results:**

About 41.7% of children were stunted. Children aged 36–47 months [AOR 6.22; 95% CI (1.81–21.36)], and 48–59 months [AOR 7.27; 95% CI (1.22–43.19)], sex of the child [AOR 20.79; 95% CI (7.50–57.65)], birth order [AOR 6.42; 95% CI (1.68–24.48)], mother education [AOR 0.06; 95% CI (0.02–0.14)], having toilet facility [AOR 0.059; 95% CI (0.02–0.18)], washing hand by soap [AOR 16.21; 95% CI (5.11–51.4)] and ANC [AOR 0.045; 95% CI (0.01–0.13)] were associated with stunting.

## Introduction

Malnutrition is one of the most common causes of illness and death among children throughout the world. It has been accountable directly or indirectly for 60% of the 10.9 million deaths yearly among under-5 children [1, 2]. Studies conducted in various parts of Ethiopia displayed the magnitude of stunting between 35.4 and 47.6% [3–7]. In South Ethiopia, 44% children are stunted [8].

The political and financial circumstances, education, climate, culture, breast-feeding, custom of food are some of the features that affect nutritional status of a given community [9]. Factors that can affect nutritional status of the children could be age [4, 6, 7], being male child [3, 4], mothers education [7, 8] and attending antenatal care (ANC) [10–12]. Availing data on stunting level and its determinants is important for preparation and employment of interventions that focus on reducing illness and death associated with stunting. Therefore, this paper focused on examining the magnitude and predictors of stunting among children of age 6–59 months in rural parts of Damot Gale.

## Main text

### Methods

#### Study area and study period

Our study was done in rural part of Damot Gale district. Damot Gale district is located in Wolayita zone, southern Ethiopia. The total areal size of the Damot gale district is 255.54 km^2^ [13]. Geographically, the district is situated between the coordinate of 6°32′24″N and 7°7′30″N latitude and 37°44′53″E and 37°56′24″E of longitude. In 2013, the total predicted population of Damot Gale is 186,687. About 22,069 children aged 6–59 months are living in rural parts of the district. The study was conducted in February 2016.

#### Study design

Community based cross-sectional.

#### Study population

Children aged 6–59 months and their mothers.

#### Sample size and sampling technique

The sample size was computed by single population proportion formula.

$${\text{n}} = \frac{{\left( {{\text{Z}}_{{{\alpha }/2}} } \right)^{2} }}{{{\text{d}}^{2} }}[{\text{p}}(1 - {\text{p}})]$$

where n = sample size required, p = estimated proportion of children aged 6–59 months with stunting. z = Z-score associated with appropriately chosen level of confidence, d = acceptable margin of errors.

To compute the sample size, the prevalence of stunting, 44.1% was taken from study of south Ethiopia [8]. The margin of error of 5% and 95% level of confidence was also considered. Adding 5% contingency for expected non-response rate, the final sample size became 398.

A two-phase cluster sampling design was employed. The first sampling element was Kebele (small administrative unit) and the second sampling elements were households (HH) who have children aged 6–59 months along with their mothers. At first stage of sampling, simple random sampling process was employed to select Kebele. Then, the HH of the designated kebele was gotten from the health extension employees. The number of worthy of being chosen HH was assigned based on population number using proportional allocation method. In case where, HH has more than one worthy of being chosen child, one child was designated by lottery system.

#### Data collection processes, measurements and definitions

The data collection instrument was set with allusion to the 2011 Ethiopian demographic and health survey (EDHS). It was first set in English, interpreted to wolayia then, re-interpreted back to English to ensure uniformity.

Two days training was given for data collectors and supervisors. Pre-test was made on similar eligible study children from the adjacent enumeration part. Calibrated gauging board was used to measure the height of the children. A recumbent position was used to measure height of the children who cannot stand erected. The child who can stand erected and above 24 months was measured on standup position against calibrated height gauging board. The measurement was taken to the adjacent 0.1 cm. To maintain the quality of data, the investigator and supervisors supervised the data collection area during the entire period of data collection.

Stunting (height for age) was the dependent variable of the study. The independent variables included socio-demographic and economic feature such as a child’s age and sex, family size, birth order and interval, HH income source, marital status, education and occupation of mother. Maternal health feature composed of attending ANC, giving birth at health institution and using contraceptive. Child health features were exclusive breastfeeding, health situation, existence of diarrhea, and vaccination, eating with older sibling. On environmental health situation: hand washing, cleanliness during feeding and food preparation, presence of latrine and sources of water were collected. Stunted was defined based on Z-score below − 2 standard deviations of the WHO median reference for height-for-age.

#### Statistical analysis

Data were entered and analyzed by SPSS version 20. Age of child, sex, and height were entered into World Health Organization (WHO) Anthro software to create measurement indices of height-for-age. Data recorded in WHO Anthro was transferred to SPSS version 20. Binary logistic regression analysis was carried out at two levels. First, a bivariate analysis was done to assess the predictors of stunting by explanatory variables. Secondly, those predictor variables, which are significantly associated with the outcome variable at 0.2 and less level of significance in the bivariate analyses, were examined at multivariate logistic regression. To display strength of association between dependent and independent variables, odds ratio at 95% CI were computed. Variables with p-value < 0.05 in multivariate analyses were used to state statistical significance.

### Results

#### Socio-demographic and economic features

The study comprised 398 children aged 6–59 months. About 33.7% of children were in the age range of 36–47 months. Regarding sex of the children, 43% were males. The big percentage (91.7%) of mothers were currently married. Above half of the mothers (56%) were illiterate. Forty-six (11.6%) of women had secondary and above secondary education level. Agriculture was the leading source of HH income for 340 (85.4%) respondents. First births constituted 56 (14.1%) and 5+ birth orders constituted 155 (38.9%). About 295 (71.1%) HH has one child. About 214 (53.8%) HH agricultural land was less than half a hectare. The main source of water for HH was public pipe, 287 (72.1%). Concerning about toilet facilities, 358 (90%) HH had latrine. Non-improved pit latrine was used in 276 (77.1%) HH (Table 1) [14].

**Table 1:** Socio demographic and economic features of the respondents, Damot Gale, 2016

Features (n = 398)	Categories	Number	Percent
Age of child in months	6–11	36	9.0
	12–23	113	28.4
	24–35	79	19.8
	36–47	134	33.7
	48–59	36	9.0
Child’s sex	Male	171	43.0
	Female	227	57.0
Current marital status of mothers	Married	365	91.7
	Separated	17	4.3
	Divorced	2	0.5
	Widowed	14	3.5
Educational status of mother	Illiterate	223	56.0
	Primary	129	32.4
	Secondary and above	46	11.6
Source of HH income	Agriculture	340	85.4
	Off-farm	31	7.8
	Non-farm	27	6.8
Birth order	One	56	14.1
	Two to three	73	18.3
	Four to five	114	28.6
	Above five	155	38.9
Birth interval in months	First birth	56	14.1
	Less than 24	127	31.9
	24–35	107	26.9
	36–47	61	15.3
	48 and above	47	11.8
Number of children aged 6–59 months in HH	One	295	74.1
	Two to three	103	25.9
Family size	Two to four	75	18.8
	Five to eight	284	71.4
	Nine to eleven	39	9.8
Agricultural land size in hectares	Less than half	214	53.8
	Half to one	116	29.1
	Greater than one and less than two	58	14.6
	Greater than two	10	2.5
Situations child’s mother routinely wash her hand by using soap	Before nourishing the baby	90	21.9
	Before breast nursing	37	9.0
	After washing baby’s bottom	160	38.9
	Before food preparation	124	30.2
Type of latrine	Pit	276	77.1
	Improved	82	22.9
Source of water for drinking	River	31	7.8
	Public pipe	287	72.1
	Spring	80	20.1

#### Maternal and child health utilization

About 167 (42%) children aged less than 6 months were not exclusively breastfed. Three hundred seven (81%) children were fully vaccinated. less than half, 141 (35.4%) of mothers did not received ANC service. Only 109 (27.4%) of women gave birth at health institution. More than half, 235 (59%) mothers used contraceptive. Fifty-six (14%) children had diarrhoea (Table 2) [14].

**Table 2:** Maternal and child health service features, Damot Gale, 2016

Features	Categories	Number	Percent
Fully vaccinated	No	72	19.0
	Yes	307	81.0
Exclusive breast feeding	No	167	42.0
	Yes	231	58.0
Eat with older sibling	No	364	91.5
	Yes	34	8.5
Followed ANC	No	141	35.4
	Yes	257	64.6
Place of delivery	Health institution	109	27.4
	Home	289	72.6
Used contraceptive	No	235	59.0
	Yes	163	41.0
Children health situation	Always sick	20	5.1
	Sometimes sick	108	27.1
	Healthy	270	67.8
Diarrheal happen	No	342	86.0
	Yes	56	14.0

#### Magnitude of stunting

The study revealed that 41.7% of children were stunted.

#### Predictors of stunting

On multivariate analysis, age of child, sex, birth order, mother education, washing hand, availability of latrine and ANC were associated with stunting. Children aged 36–47 months and 48–59 months were 6.22 and 7.27 times more probable to be affected by stunting as compared to children whose age was 6–11 months [AOR 6.22; 95% CI (1.81–21.36)], AOR 7.27; 95% CI (1.22–43.19). Male children were 20.8 times more to be affected by stunting as compared to female children [AOR 20.79; 95% CI (7.50–57.65)]. Birth order above five were found 6.42 times more probable to be stunted than first born children [AOR 6.42; 95% CI (1.68–24.48)]. Children whose mothers have a primary education were 94% less probable to have stunted children than illiterate mothers [AOR 0.06; 95% CI (0.02–0.14)]. Having toilet facility reduced the chance of stunting by 94% [AOR 0.059; 95% CI (0.02–0.18)].

Children who did not usually wash their hand by using soap before eating and after defecating were 16.21 times more to be stunted than children who did [AOR 16.21; 95% CI (5.11–51.40)]. Mothers followed ANC had 95.5% less probable to have stunted children than mothers did not [AOR 0.04; 95% CI (0.01–0.13)] (Table 3).

**Table 3:** Predictors of stunting, Damot Gale, 2016

Features	Categories	Stunting			
		No (N, %)	Yes (N, %)	COR at 95%	AOR at 95% CI
Age of child in months	6–11	30 (12.9)	6 (3.6)	1.00	1.00
	12–23	83 (35.8)	30 (18.1)	1.28 (0.318–5.21)	1.22 (0.34–4.42)
	24–35	52 (22.4)	27 (16.3)	6.24 (1.18–32.78)*	3.96 (0.91–17.33)
	36–47	46 (19.8)	88 (53.0)	9.46 (2.08–43.1)*	6.22 (1.81–21.36)*
	48–59	21 (9.1)	15 (9.0)	18.25 (2.58–128.8)*	7.27 (1.22–43.19)**
Sex of child	Female	171 (73.7)	56 (33.7)	1.00	1.00
	Male	61 (26.3)	110 (66.3)	32.38 (9.89–106.11)*	20.80 (7.50–57.65)*
Birth orders	First child	45 (19.4)	11 (6.6)	1.00	1.00
	2–3	62 (26.7)	11 (6.6)	0.894 (0.16–4.99)	0.70 (0.14–3.38)
	4–5	72 (31.1)	42 (25.3)	1.43 (0.366–5.56)	1.28 (0.36–4.51)
	5+	53 (22.8)	102 (61.5)	8.12 (1.93–34.19)*	6.42 (1.68–24.48)*
Educational status of mother	Illiterate	75 (32.4)	148 (89.2)	1.00	1.00
	Primary	120 (51.7)	9 (5.4)	0.06 (0.02–0.17)*	0.06 (0.02–0.14)*
	Secondary and above	37 (15.9)	9 (5.4)	0.34 (0.06–1.98)	0.32 (0.06–1.55)
Availability of latrine	No	6 (2.6)	36 (21.7)	1.00	1.00
	Yes	226 (97.4)	130 (78.3)	0.028 (0.01–0.14)*	0.06 (0.02–0.18)*
Child usually wash her/his hand’s by using soap	Before eating	96 (41.4)	40 (24.1)	1.00	1.00
	After defecating	32 (13.8)	14 (8.4)	1.12 (0.197–6.33)	0.95 (0.203–4.45)
	Both	39 (16.8)	4 (2.4)	0.12 (0.025–0.621)*	0.18 (0.05–0.72)**
	None of the above	65 (28.0)	108 (65.1)	24.89 (7.13–86.84)*	16.21 (5.11–51.4)*
Following ANC	No	36 (15.5)	105 (63.3)	1.00	1.00
	Yes	196 (84.5)	61 (36.7)	0.11 (0.012–0.795)*	0.045 (0.01–0.135)*

* p-value < 0.2, ** p-value < 0.01, *** p-value < 0.05

### Discussion

This study tried to assess magnitude of stunting and associated factor among 6–59 months old children. Based on the findings the study, the magnitude of stunting was 41.7%. This is somewhat comparable with study of EDHS and Hawassa Zuria, which presented stunting as 44.1 and 45.8%, respectively [5, 8]. Generally, magnitude of stunting in the study area are higher compared to the cutoff point stated by Federal Minister of Health [15]. The magnitude of stunting in current study is lower than study of Oromia region, which displayed 47.6% of children were stunted [6]. But, it is higher than study conducted in Somalia region and Hossana town, which showed 34.4 and 35.5% of children were stunted, respectively [3, 7]. The difference may be due to difference in study setting, difference in child feeding habit in rural and urban settings.

The result of the present study showed that child’s age is one of determinant feature of stunting. This result is in line with studies conducted in Ethiopia and other developing countries [6, 7, 16, 17].

The finding of this study revealed that male children were more affected by stunting as compared to female children. This is consistent with results of West Gojam and other studies [3, 18, 19].

The current study showed that the risk of stunting increases when birth order was above 5. This finding agrees with study conducted in Uganda [20]. It also aggresses with study conducted in Hossana [7]. The positive significant relationship between child birth order and occurrence of stunting could be explained by the fact that the family pot is shared among a huge number of people in the HH, thus inadequate dietary can be in taken by children for a prolonged period and eventually the manifestation of chronic malnutrition starts [21]. The current study is incomparable with study done in Ethiopia, which showed first birth order were found to be at a considerably higher risk of stunting than higher birth order [16].

Children whose mothers have a primary level of education had a significantly reduced likelihood of stunting as compared to illiterate mothers. This is consistent with study done in Hossana and by EDHS [7, 8]. This may be due to education is one of the most important resources that enable women to provide appropriate care for their children. Educating women is believed to exert an impact on health and nutritional status of children since it provides the mother with the necessary skills for childcare, increase awareness of nutritional needs and preference of modern health facilities.

Having toilet facility reduced the chance of stunting. This is in line with the results of other study [20]. Another study conducted in Nigeria also revealed that having toilet has a significant negative effect on the chance of stunting [19]. The reason for the above studies can be explained by the following sentences. Unfavorable health environment can increase the probability of infectious diseases and indirectly can cause certain types of malnutrition.

Children who did not usually wash their hand by using soap before eating and after defecating were more probable to have had stunting than children who did. This is not surprising since there is a relationship between sanitation and infectious diseases. Study conducted on children revealed proper hand washing before meals and after defecating, could lower exposure to germs. This can lessen the chances of illness and chronic inflammation leading to better nutrition intake, more energy available for growth and development [22].

Following ANC was associated with stunting. This is consistent with other studies conducted in Ethiopia and other developing countries [10–12].

The current study shows that stunting is a common among children 6–59 months age. Child’s sex, birth order, following ANC, age of child, mothers’ education, washing hand, and availability of latrine were predictors of stunting.

Equivalent consideration should be given during feeding both male and female children. Promotion of ANC should be strengthened especially in rural setting. The health extension workers and other concerned body should do more on increasing availability of latrine and hand washing facilities. Health education on importance of hand washing should be strengthened at community level. Educational difference has an impact on child stunting, so due attention should be given to promote and increase education coverage of females.

## Limitation of the study

Questions that required a good memory were vulnerable to recall bias.

As the study is cross-sectional, it neither characterises seasonal difference of nutritional outcomes nor creates causal association.

## Abbreviations

ANC: antenatal care
EDHS: Ethiopian demographic health survey
HH: households
WHO: World Health Organization
